# Effects of royal jelly consumption on inflammation and oxidative stress: A systematic review and meta-analysis of randomized controlled trials

**DOI:** 10.22038/ajp.2024.25139

**Published:** 2025

**Authors:** Shaghayegh Taheri, Hossein Bahari, Farshad Mirzavi, Pegah Rahbarinejad, Zohreh Sajadi Hezaveh, Armin Doostparast, Asghar Zarban, Elyas Nattagh-Eshtivani

**Affiliations:** 1 Department of Clinical Biochemistry, School of Medicine, Birjand University of Medical Sciences, Birjand, Iran; 2 Department of Nutrition, Faculty of Medicine, Mashhad University of Medical Sciences, Mashhad, Iran; 3 Transplant Research Center, Clinical Research Institute, Mashhad University of Medical Sciences, Mashhad, Iran; 4 Cardiovascular Diseases Research Center, Birjand University of Medical Sciences, Birjand, Iran; 5 Faculty of Medicine and Health, Department of Health Sciences, The University of Sydney, Sydney, NSW, Australia; 6 Department of Nutrition, Food Sciences and Clinical Biochemistry, School of Medicine, Social Determinants of Health Research Center, Gonabad University of Medical Science, Gonabad, Iran

**Keywords:** Malondialdehyde, Antioxidant, Royal jelly, Oxidation-reduction, C-reactive protein, Meta-analysis

## Abstract

**Objective:**

This systematic review and meta-analysis examines the impact of royal jelly (RJ) on inflammation and oxidative stress. By synthesizing existing research, it aims to provide valuable insights into the potential health benefits of RJ.

**Materials and Methods:**

PubMed/Medline, Web of Science, and Scopus were searched until the end of December 2023. This meta-analysis included all randomized clinical trials assessing the effect of RJ supplements on serum levels of high-sensitivity C-reactive protein (hs-CRP), total antioxidant capacity (TAC), and malondialdehyde (MDA). A random-effects model was utilized to calculate the pooled mean differences (MD) and 95% confidence interval.

**Results:**

Seven suitable datasets from 6 trials were considered eligible. RJ supplementation significantly reduced MDA (WMD, –1.79 (–3.00 to –0.58), p=0.004; I^2^ = 97.4%) and increased TAC (WMD, 0.98 (0.24 to 1.71), p=0.009, I^2^ = 98.5%), but it did not significantly change hs-CRP levels (WMD: -0.24; 95% CI: -0.60, 0.10; p=0.17). RJ supplementation in higher doses and in participants with normal body mass index (BMI) could induce a greater elevation in TAC, and in participants with normal BMI, a stronger reduction in MDA.

**Conclusion:**

Although this meta-analysis confirmed that RJ could be a useful intervention to reduce oxidative stress, this research should be updated in future due to the restricted number of trials.

## Introduction

Among various definitions of oxidative stress, the simplest and most popular definition presented by Sies in 1985 is “an imbalance between oxidant and antioxidants potentially leading damage” (Sies, 1985). Indeed, the overproduction of reactive oxygen species (ROS) may be able to destroy antioxidant defense system and deteriorate normal status (Pizzino et al., 2017). 

Reactive oxygen species, as active forms of oxygen, are essential for cellular function such as rest and reaction to various stimuli (Valko et al., 2007). ROS play key roles in major signaling pathways, including changes in cell physiology through transcription or posttranscriptional modulation following transduction of extracellular stimuli (Bahari et al., 2023a). Moreover, redox signaling pathway has vital roles such as the production of NO•, the control of vascular constriction and relaxation, transmission of nerve signals, cellular attachment, immune system reaction, and the sensing of hypoxia and cell death (Dröge, 2002; Valko et al., 2007).

Despite the aforementioned physiological roles of oxidative stress, its pathogenic roles contribute to etiology of multiple chronic conditions, such as cancer, cardiovascular diseases (CVDs), neurological disease, diabetes, respiratory disease, rheumatoid arthritis, and kidney diseases (Pizzino et al., 2017). Furthermore, oxidative stress can be measured by biomarkers (Dalle‐Donne et al., 2005). For instance, as an indirect assessment of oxidative stress, 8-hydroxydeoxyguanosine (8-OHdG), Malondialdehyde (MDA), and 3-nitrotyrosines are most commonly used biomarker for DNA/RNA damage, oxidation of lipids and the oxidation/nitration of proteins, respectively (Ho et al., 2013).

To counteract ROS, human body adopts different strategies using antioxidant molecules that can be categorized into enzymatic (such as superoxide dismutase (SOD), catalase (CAT), and glutathione peroxidase (GPx)) and non-enzymatic forms (e.g. lipoic acid, glutathione, ʟ-arginine, and coenzyme Q10). All of above agents are called endogenous antioxidants. Besides, vitamin E, flavonoids, ascorbic acid, and polyphenols are exogenous antioxidant molecules that originate from diet or nutritional supplementations (Pizzino et al., 2017). A growing body of evidence shows that royal jelly (RJ) with a wide array of pharmacological effects and antioxidant properties has a protective effect against oxidative stress in human beings (Kamakura and Sakaki 2006; Petelin et al., 2019; Pourmoradian et al., 2014; Shidfar et al., 2015; Watanabe et al., 1998). 

Royal jelly is a milky and viscous mixture produced and released by the hypopharyngeal and mandibular glands of nurse honeybees (*Apis mellifera*) (Šimúth 2001; Zahmatkesh et al., 2014). It is substantial for queen reproduction and the growth of larvae. RJ mainly contains water (60–70%), and three major macronutrients including carbohydrates (10–12%), proteins (12–15%), and lipids (3–7%) (Suzuki et al., 2008). In addition to the antioxidant property, previous studies indicated that this functional food has anti-inflammatory (Kohno et al., 2004; Pourmobini et al., 2021), vasodilative and hypotensive activity (Tokunaga et al., 2004), anti-hypercholesterolemic (Bahari et al., 2023b; Guo et al., 2007), anti-tumor (Townsend et al., 1959), and hypoglycemic functions (Bahari et al., 2023c; Khoshpey et al., 2016). Furthermore, anti-oxidant and anti-inflammatory properties of RJ are attributed to 10-hydroxy-2-decenoic acid (10-HDA), as the primary bioactive element of utmost significance (Honda et al., 2015). Based on the available information, there is no structured and comprehensive review and meta-analysis of the effect of RJ on inflammation and oxidative stress. Therefore, the aim of the present study was to pool the existing evidence regarding the effect of RJ on inflammation and oxidative stress.

## Materials and Methods

The Preferred Reporting Items for Systematic Reviews and Meta-analysis (PRISMA) guidelines were adhered to (Moher et al., 2015).

### Search strategy

To gather the references for this review, searches were conducted on PubMed/Medline, Web of Science, and Scopus until the end of December 2023. The following terms and keywords were used for the search: ("royal jelly") AND (intervention OR intervention* OR "controlled trial" OR randomized OR randomised OR random OR randomly OR placebo OR "clinical trial" OR trial OR "randomized controlled trial" OR "randomized clinical trial" OR rct OR blinded OR "double blind" OR "double blinded" OR "clinical trials" OR trials OR "Cross-Over" OR parallel). The search did not have language restrictions. Furthermore, we manually examined all of the references included in the included articles to avoid missing any relevant studies.

### Inclusion and exclusion criteria

PICOS model was used for eligibility criteria that are abbreviation of participants (adults aged>18 years), intervention (royal jelly), comparator (placebo), outcome (including high sensitivity C-reactive protein (hs-CRP), total antioxidant capacity (TAC) and malondialdehyde (MDA)) and study design (parallel and crossover clinical trial) (Richardson et al., 1995).

Studies were considered for inclusion if they met these criteria: 1) randomized clinical trials (RCTs) comparing the eﬀect of royal jelly supplementation to placebo; 2) included adult participants (>18 years old); 3) reported mean and standard deviation (SD) or standard error (SE) of parameters; 4) the treatment group exclusively received royal jelly as an active intervention compared to a control group, and there were no other interventions given in combination with it. Studies were excluded if they were: 1) not randomized or without a group served as a control; 2) insufficient data for statistical pooling; 3) if they were in combination with other interventions. The current research excluded observational, or non-interventional studies, animal studies, reviews, comments, books, letters, and conference papers with no full-text available.

 H.B. did the search. Two authors (H.B. and Sh.T.) screened all titles and abstracts separately and acquired the full text of every paper that was certainly or probably eligible. Then the two authors checked the articles in full text against the eligibility criteria. E.N.E. resolved any disagreement that arose between the two authors.

### Data extraction

Data according to characteristics of the studies that were included authors, year, country, study design, trial duration, number of participants, body mass index (BMI), and intervention details; participant details (age, sex and health status); outcomes assessed, including baseline and ﬁnal outcome measures (hs-CRP, TAC, and MDA) were extracted by two authors (H.B. and Sh.T.), who also evaluated the quality level of eligible studies and cross-checked the data. If additional data was needed, we reached out to authors of articles. If necessary, any disparities or inconsistencies were resolved by the third investigator (E.N.E.).

### Quality assessment of studies

To evaluate the reporting quality of the eligible studies, Cochrane's tool for the risk of bias assessment in randomized trials was used (Higgins et al., 2011). Two authors assessed the quality of all the RCTs included (H.B. and Sh.T.) for the following domains: Random sequence generation, allocation concealment, selective reporting, other sources of bias, blinding of participants and personnel, blinding of outcome assessment, incomplete outcome data, and general risk of bias. Studies were rated as low (L), or high risk of bias (H) or unclear (U) for each bias category, according to the Cochrane Handbook's guidelines (Moher et al., 2010). The GRADE methodology was used to assess the quality and value of the body of retrieved evidence (Guyatt et al., 2011).

### Statistical analysis

The effect size was calculated using the mean change and SD for hs-CRP, TAC, and MDA measurements in both groups of intervention and placebo. For studies with no reported SD of the mean difference, the following formula was used (Hozo et al., 2005): 

SD = √n (values within group) × SEM. 

Mean differences (MDs) and 95% conﬁdence intervals (CIs) of studies were calculated by pooling baseline and ﬁnal mean and standard deviation (SD) values of the studies in both intervention and comparison groups. The random effect model was used. We also conducted sensitivity and subgroup analyses. The heterogeneity and diversity among the studies was evaluated utilizing the Q test employing a significance level of p<0.10, and the I² statistic was used to quantify it. A clinically important heterogeneity was set at I^2^ >75% (DerSimonian and Kacker 2007). Subgroup analysis was carried out to identify potential causes of heterogeneity between studies, focusing on the intervention dosage, participant health status, sex, and baseline BMI. To determine the potential origin and effect of any bias, sensitivity analysis was conducted. Publication bias was evaluated using the Begg’s test, and the Egger’s regression test. Funnel plots were also visually examined (Egger et al., 1997b). The process of analyzing statistics and synthesizing data was executed using Stata software, version 17.0 (Stata Corp LP, College Station, TX, USA).

## Results

### Study selection

**Figure 1:**
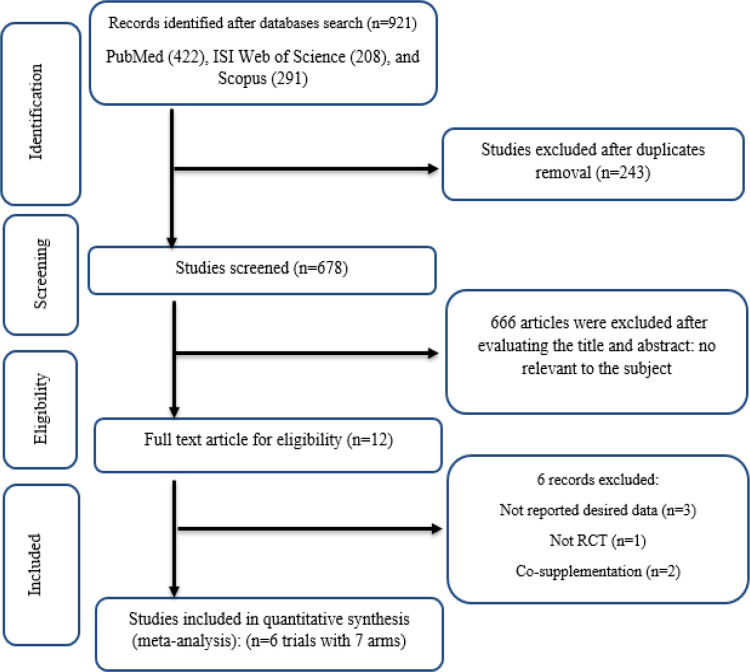
Flow chart of study selection for inclusion trials in the systematic review

After searching the internet-based databases of PubMed, Scopus, and Web of Sciences, 921 articles were initially found, from which 243 studies were excluded due to duplication, and 666 articles due to irrelevancy following the inclusion criteria, were excluded. The full texts of the 12 studies were read, which led to the exclusion of papers due to not reporting the desired data (n=3) (Ab Wahab et al., 2018; Araki et al., 2018; Asama et al., 2018), not RCT (n=1) (romouzi 2023), and combination therapy (n=2) (Abedini et al., 2021; Zendelovska et al., 2023). Finally, 6 studies with 7 treatment arms were incorporated into our meta-analysis (Figure 1).

### Study characteristic

Features and hallmarks of the included studies are presented in Table 1. This meta-analysis was performed by including six articles published between 2014 and 2023 with 356 participants (181 interventions and 175 controls). The intervention duration of all included trials was 8 weeks, except for the intervention by Fujisu et al (Fujisue et al., 2022) which lasted for 4 weeks, and the intervention dose was from 1000 to 5000 mg per day. The sample size varied between 40 and 88 people, and all studies incorporated parallel RCTs designs. The participants in the studies were either asymptomatic overweight individuals (Petelin et al., 2019), patients with addiction (Sargazi and Taghian 2023), patients with type 2 diabetes mellitus (T2DM) (Mobasseri et al., 2014; Pourmoradian et al., 2014; Shidfar et al., 2015), or healthy adults (Fujisue et al., 2022). The reviewed studies were conducted in Iran (Mobasseri et al., 2014; Pourmoradian et al., 2014; Sargazi and Taghian 2023; Shidfar et al., 2015), Japan (Fujisue et al., 2022), and Slovenia (Petelin et al., 2019). Two studies included only female participants (Mobasseri et al., 2014; Pourmoradian et al., 2014), one included only male participants (Sargazi and Taghian, 2023), and the other three included both sexes (Fujisue et al., 2022; Petelin et al., 2019; Shidfar et al., 2015).

**Table 1:** Characteristic of the studies included in meta-analysis

**Studies**	**Country**	**Study Design**	**Participant**	**Sex**	**Trial Duration** **(Week)**	**Total Sample size**	**Sample size**		**Means Age**		**Means BMI**		**Intervention**			**Outcome(s)**
							**IG**	**CG**	**IG**	**CG**	**IG**	**CG**	**Type of intervention**	**dose (mg/d)**	**Control group**	
Pourmoradian et al. 2014	Iran	R, DB, Parallel	T2DM	F	8	41	21	20	51.7±6.3	51.4±9.6	28.98±1.1	28.8±1.3	RJ soft gel	1000	Placebo	RJ supplementation significantly reduced MDA. The mean TAC elevated insignificantly in both groups.
Mobasseri et al. 2014	Iran	R, DB, Parallel	T2DM	F	8	41	21	20	51.7±6.3	51.4±9.6	28.98±1.1	28.8±1.3	RJ soft gel	1000	Placebo	hs-CRP level was reduced in the supplemented group whereas it was elevated in the placebo group; however, these changes were not significant.
Shidfar et al. 2015	Iran	R, DB, Parallel	T2DM	B	8	46	23	23	51.8	53.1	27.79±2.6	28.01±3.8	RJ capsule	3000	Glycerin	Serum TAC increased significantly in RJ group. The changes in MDA levels was not significant.
Petelin et al. 2019	Slovenia	R, DB, Parallel	Asymptomatic Overweight Adults	B	8	60	30	30	41.1±11.8	41.1±7.4	26.2±1.7	27.7±3.7	RJ capsule	2000	Placebo	RJ supplementation demonstrated a significant decrease in CRP levels, whereas a significant increase was observed in TAC.
Fujisue et al. 2022	Japan	R, DB, Parallel	Healthy adult	B	4	88	46	42	36.1±9.1	35±9.3	22.3±2.4	22.4±3	RJ tablet	2040	Placebo	hs-CRP level remained unchanged after RJ supplementation.
Sargazi et al. 2023 (a)	Iran	R, Open, Parallel	Addicts	M	8	40	20	20	35.6	36.5	22.03±2.1	20.63±1.1	RJ capsule	5000	Nothing	MDA levels significantly decreased after RJ supplementation compared to the control group. Also, a significant increase in TAC was observed.
Sargazi et al. 2023 (b)	Iran	R, Open, Parallel	Addicts	M	8	40	20	20	37.3	34.8	21.74±1.1	21.43±2.1	RJ capsule	5000	Exercise	MDA levels significantly decreased after RJ supplementation + exercise compared to the exercise group. Also, a significant increase in TAC was observed.

Abbreviations: IG, intervention group; CG, control group; DB, double-blinded; R, randomized; T2DM, type 2 diabetes mellitus; F, Female; M, Male; B, Both sexes; RJ, Royal Jelly; hs-CRP, High-sensitivity C-reactive protein; MDA, malondialdehyde; TAC, total antioxidant capacity

### Meta-analysis

#### Effects of royal jelly supplementation on oxidative stress

**Figure 2:**
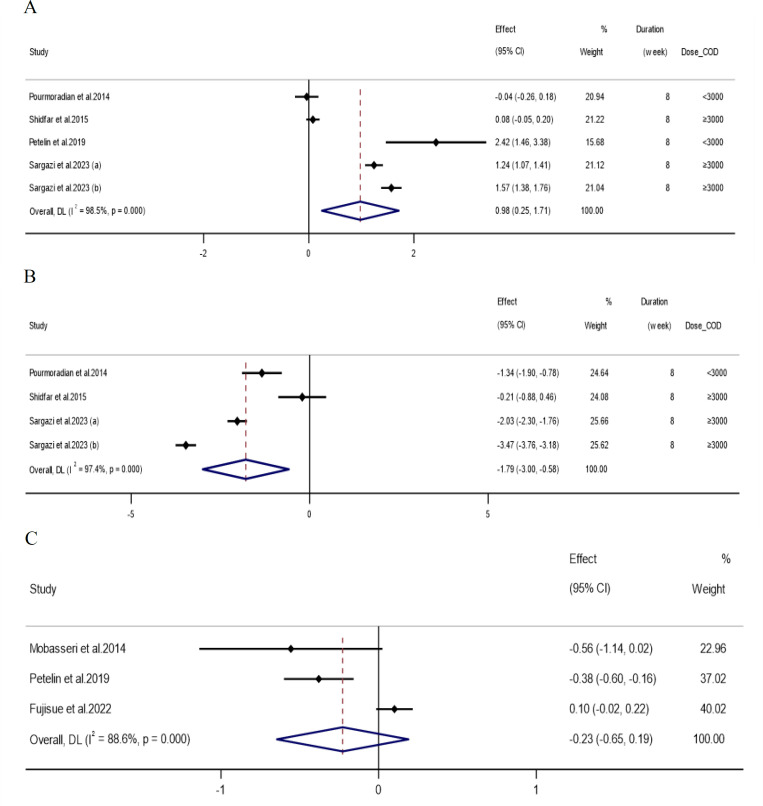
Forest plot detailing weighted mean difference and 95% confidence intervals (CIs) for the effect of royal jelly intake on A) TAC (mmol/L); B) MDA (nmol/ml); and C) hs-CRP (mg/L)

Table 3 summarizes the all-study and subgroup analysis of the impact of royal jelly on hs-CRP, MDA and TAC, and Figure 2 presents the visual results (subgroup analysis was not performed for hs-CRP, due to the limited number of eligible articles). Analysis of 5 overall effect sizes for TAC (Petelin et al., 2019; Pourmoradian et al., 2014; Sargazi and Taghian 2023; Shidfar et al., 2015) and 4 overall effect sizes for MDA (Pourmoradian et al., 2014; Sargazi and Taghian 2023; Shidfar et al., 2015) showed significant increases in TAC (WMD: 0.98; 95%CI: 0.24,1.71; p=0.009) and significant decreases in MDA (WMD: -1.79; 95% CI: -3.00, -0.58; p=0.004). Royal jelly supplementation with a dose ≥3000 (WMD: 0.96; 95%CI: 0.01, 1.91; p=0.048) or supplement consumption in participants with normal BMI (WMD: 1.40; 95%CI: 1.07, 1.72; p=0.001) caused a significant increase in TAC. Also, royal jelly supplementation either with a dose ≥3000 or <3000 mg/day ((≥3000: WMD: -1.93; 95%CI: -3.39, -0.47; p=0.009), (<3000: WMD: -1.34; 95%CI: -1.89, -0.78; p=0.001)) and in participants with normal BMI (WMD: -2.74; 95%CI: -4.16, -1.33; p=0.001) significantly reduced MDA. Subgroup analysis showed that the level of both factors changed significantly in non-diabetic participants ((TAC: WMD: 1.52; 95%CI: 1.15, 1.89; p=0.001), (MDA: WMD: -2.74; 95%CI: -4.16, -1.33; p=0.001)).

### Quality assessment

In terms of the overall risk of bias, 4 studies with low risk (Mobasseri et al., 2014; Petelin et al., 2019; Pourmoradian et al., 2014; Shidfar et al., 2015) and 2 of them with moderate risk (Fujisue et al. 2022; Sargazi and Taghian 2023) were evaluated (Table 2). In addition to other sources of bias (Fujisue et al., 2022; Sargazi and Taghian 2023), high risk of bias was detected in 2 studies due to selective reporting (Fujisue et al., 2022; Mobasseri et al., 2014), and 1 study due to blinding of participants and personnel (Sargazi and Taghian 2023).

### Effects of royal jelly supplementation on CRP

Three out of 6 studies reported the effect of royal jelly on hs-CRP (Fujisue et al. 2022; Mobasseri et al. 2014; Petelin et al. 2019). Although this effect was found to be non-significant (WMD: -0.24; 95% CI: 0.60, 0.10; p=0.17), no conclusive judgment could be reached due to the limited number of included studies in this meta-analysis.

Between-study heterogeneity (I^2^) ranged from 87.2% for hs-CRP to 99.5% for TAC and did not decrease significantly in different subgroups.

**Table 2:** Risk of bias assessment of the included studies

**Study**	**Random sequence generation**	**Allocation concealment**	**Selective reporting**	**Blinding (participants and personnel)**	**Blinding (outcome assessment)**	**Incomplete outcome data**	**Other sources of bias**	**General risk of bias**
Pourmoradian et al.2014	L	L	L	L	L	L	L	L
Mobasseri et al.2014	L	L	H	L	L	L	L	L
Shidfar et al.2015	L	L	L	L	U	L	L	L
Petelin et al.2019	L	L	L	L	L	L	L	L
Fujisue et al.2022	L	L	H	L	L	L	H	M
Sargazi et al.2023	L	U	L	H	L	L	H	M

L, Low;U, unclear; H, high. general low risk < 2 high risk. general moderate risk = 2high risk. general high risk > 2 high risk

### Sensitivity analysis

To evaluate the effect of each study separately on the collective effect size, we checked whether the overall effect would change if each of the studies were excluded. The overall effect size was significantly affected after removing Petelin et al. 2019 trial for TAC (WMD: 0.71, 95%CI: -0.07, 1.49) and Sargazi 2023(a) trial for MDA (WMD: -1.68, 95%CI: -3.72, 0.34). leaving the study by Fujisu et al out of the meta-analysis led to a significant decrease in hs-CRP following royal jelly supplementation (WMD: -0.40, CI95%: -0.60, -0.19).

### Publication bias

**Figure 3:**
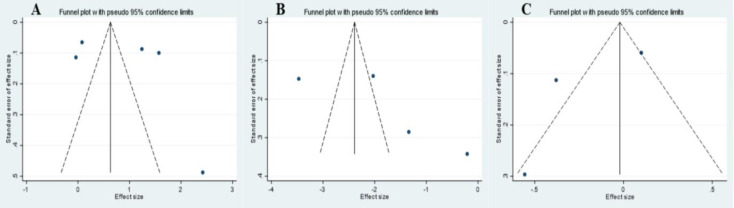
Funnel plots for the effect of royal jelly intake on A) TAC (mmol/L); B) MDA (nmol/ml); and C) hs-CRP (mg/L).

Begg’s and Egger’s tests were conducted, and the results of both tests showed that there was no bias in publication. Observing the funnel plot diagrams (Figure 3), it is apparent that most dots are fallen outside of the funnel. This observation not being in line with the result of Egger’s and Begg’s test, could be due to chance, data irregularities, or true heterogeneity (Egger et al., 1997a). 

### GRADE analysis

We used the GRADE method to determine the overall quality of the evidence. The Moderate quality of evidence was observed in the evaluation of the complementary outcome of RJ consumption on MDA and TAC factors; However, the quality of existing evidence in regard to hs-CRP was found to be low (Table 4).

**Table 3:** Subgroup analyses of the effect of royal jelly supplementation on hs-CRP and oxidative stress

	**NO**	**WMD (95%CI)**	**p-value**	**Heterogeneity **		
				**p heterogeneity**	**I** ^2^	**p between sub-groups**
**Royal jelly supplementation on TAC**						
Overall effect	5	0.98(0.24, 1.71)	0.009	< 0.001	99.5%	
**Intervention dose (mg/day)**						
<3000	2	1.14(-1.26, 3.55)	0.352	< 0.001	95.9%	0.889
≥3000	3	0.96(0.008, 1.91)	0.048	< 0.001	99.0%	
**Health status**						
Diabetic	2	0.04(-0.06, 0.15)	0.386	0.370	0.0%	0.001
Non-diabetic	3	1.52(1.15, 1.89)	0.001	0.005	81.3%	
**Sex**						
Both sexes	2	1.19(-1.09, 3.49)	0.306	< 0.001	95.6%	0.001
Male	2	1.40(1.07, 1.72)	0.001	0.012	84.0%	
Female	1	-0.04(-0.26, 0.18)	0.726	-	-	
**Baseline BMI (kg/m** ^2^ **)**						
Normal (18.5-24.9)	2	1.40(1.07, 1.72)	0.001	0.012	84.0%	
Overweight (25-29.9)	3	0.46( -0.08,1.005)	0.096	< 0.001	91.7%	0.004
**Royal jelly supplementation on MDA**						
Overall effect	4	-1.79(-3.00, -0.58)	0.004	< 0.001	99.3%	
**Intervention dose (mg/day)**						
<3000	1	-1.34(-1.89, -0.78)	0.001	-	-	0.456
≥3000	3	-1.93(-3.39, -0.47)	0.009	< 0.001	98.0%	
**Health status**						
Diabetic	2	-0.79(-1.89, 0.31)	0.161	0.011	84.5%	0.032
Non-diabetic	2	-2.74(-4.16, -1.33)	0.001	< 0.001	98.0%	
**Sex**						
Both sexes	1	-0.21(-0.88, 0.46)	0.539	-	-	0.002
Male	2	-2.74(-4.16, -1.33)	0.001	< 0.001	98.0%	
Female	1	-1.34(-1.89, -0.78)	0.001	-	-	
**Baseline BMI (kg/m** ^2^ **)**						
Normal (18.5-24.9)	2	-2.74(-4.16, -1.33)	0.001	< 0.001	98.0%	0.032
Overweight (25-29.9)	2	-0.79(-1.89, 0.31)	0.161	0.011	84.5%	
**Royal jelly supplementation on hs-CRP**						
Overall effect	3	-0.24(-0.60, 0.10)	0.171	< 0.001	87.2%	

**Table 4:** GRADE profile of royal jelly supplementation effect on hs-CRP and oxidative stress

Quality assessment						Quality of evidence
Outcomes	Risk of bias	Inconsistency	Indirectness	Imprecision	Publication Bias	
TAC	No serious limitations	Very Serious limitations ¹	No serious limitations	No serious limitations	No serious limitations	⊕⊕◯◯ Moderate
MDA	No serious limitations	Very Serious limitations ¹	No serious limitations	No serious limitations	No serious limitations	⊕⊕◯◯ Moderate
hs-CRP	No serious limitations	Very Serious limitations ¹	No serious limitations	Serious limitations ²	No serious limitations	⊕◯◯◯ Low

The test for heterogeneity is significant, and the I^2^ is ≥75%.2. There is no significant effect of Royal jelly consumption.

## Discussion

This meta-analysis focused on the effects of RJ supplementation on the serum levels of hs-CRP, TAC and MDA in adults. The results of this investigation revealed a considerable rise in serum TAC levels, whereas, it had no effect on hs-CRP. Also, our findings have shown a significant decrease in MDA. The observed increase in TAC levels and simultaneous decrease in MDA levels suggest a promising role of RJ in augmenting antioxidant defense mechanisms and mitigating lipid peroxidation, a hallmark of oxidative stress. These alterations align with prior evidence highlighting the potential benefits of natural products, such as RJ, in combating oxidative damage which is implicated in various pathological conditions.

The anti-oxidative efficacy of RJ is supported by these findings which also suggest that RJ may be an effective adjuvant medication that may be used along with conventional therapy to reduce oxidative stress. As previously stated, oxidative stress is thought to be an important factor in a variety of illnesses, including T2DM, CVDs, autoimmune disorders, and some kinds of cancer. (Hotamisligil 2017; Sharifi-Rad et al., 2020). The dose-dependent response observed in TAC and MDA levels underlines the importance of the administered dosage of RJ. Specifically, higher doses (≥3000 mg/day) and consumption among individuals with normal BMI exhibited more pronounced effects. These findings suggest a potential threshold effect related to RJ dosage and an interplay between body composition and the outcomes of RJ supplementation. The subgroup analysis revealed noteworthy changes in non-diabetic participants, implying a potentially greater impact of RJ on oxidative stress parameters in this specific population. This particular finding holds promise for preventive health strategies, suggesting that RJ might offer beneficial effects in non-diabetic individuals, thus warranting further exploration into the underlying biological mechanisms.

Many studies have managed to explore the effects of RJ on hs-CRP oxidative stress measurements (Petelin et al., 2019; Pourmoradian et al., 2014; Sargazi and Taghian 2023). Sargazi and Taghian (Sargazi and Taghian 2023) demonstrated that 8-week supplementation with 100 mg/kg RJ in patients under methadone maintenance therapy decreased the levels of MDA and increased TAC. In another study, Shidfar et al. indicated that 8 weeks of supplementation with 3000 mg/d of RJ increased TAC in Type 2 Diabetic Patients (Shidfar et al., 2015). However, a significant improvement was not observed in serum levels of MDA. Pourmoradian et al. demonstrated that RJ supplementation (1000 mg/day) for 8 weeks reduced MDA levels significantly without affecting TAC (Pourmoradian et al., 2014). In addition, Petelin et al. have shown that RJ supplementation caused a statistically significant decrease in inflammatory marker CRP, whereas significant increases were observed in serum levels of TAC (Petelin et al., 2019). 

When there is an abundance of free radicals and the body's antioxidant defense mechanism is unable to protect them from causing harm, oxidative stress occurs (Sargazi and Taghian 2023). In fact, when free radicals interact with lipids, they can cause lipid peroxidation, which is a major cause of cell harm (Young and Woodside 2001). Many medications and dietary supplements aim to reduce oxidative stress in order to reduce the consequences of various metabolic diseases (Camps and García-Heredia 2014). There are several findings that confirm RJ's function as a free radical scavenger in the data that is currently accessible (Guo et al., 2007; Liu et al., 2008). RJ has antioxidant characteristics since it contains organic components ranging from phenolic compounds to peptides, amino acids, and fatty acids (Boyacioglu 2022). Flavonoids are one of the subcategories of phenolic compounds, including chrysin, pinocembrin, and pinostrobin chalcone, with antioxidant properties (Boyacioglu 2022). The total phenolic content of RJ has been found to positively correlate with fluorescence recovery after photobleaching (FRAP) values (Kolayli et al., 2016). In addition to phenolic compounds, peptides, particularly three dipeptides (Lys-Tyr, Arg-Tyr, and Tyr-Tyr) with tyrosine residues at their C-termini, demonstrate potent hydrogen peroxide-scavenging capabilities. Additionally, proline and cysteine, two amino acids, are crucial for antioxidant activity. The former has the ability to scavenge hydroxyl radicals, and the latter takes part in the production of glutathione, a powerful cellular antioxidant (Nakajima et al., 2009; Silici et al., 2011). Additionally, the antioxidant action of RJ is due to hydroxy dicarboxylic fatty acids. Sebacic acid, 10-hydroxy-2-decanoic acid, and 10-hydroxy-2-decenoic acid are examples of carboxylic acids (Kocot et al., 2018). RJ contains 10-hydroxy-2-decenoic acid, which has been shown to alleviate oxidative stress. RJ has the potential to act as an efficient free radical scavenger. Additionally, RJ reduced the activity of cytochrome P450 4A14 enzymes and other detoxifying enzymes involved in the breakdown of endogenous lipid peroxidation. At the same time, it enhanced the expression of genes related to glutathione-S-transferase and glutathione peroxidases (Kanbur et al., 2009). We believe that oxidative stress and antioxidant status improvements seen in the supplemented group may be explained by these processes.

This meta-analysis holds promising strengths. The key strength of this study is that it systematically reviewed and employed a meta-analysis of RCTs to investigate the link between RJ supplementation and oxidative stress indices in adults. A further advantage of our meta-analysis is that we first assessed evidence certainty using the GRADE method. However, our research may have some limitations. First, because these indicators are measured in different ways and have different units, we were unable to evaluate the link between TAC and MDA levels, as well as the impact of RJ supplementation on any of those variables. In addition, while our recent research indicated that RJ supplementation could have positive effects on serum levels of hs-CRP, TAC and MDA in comparison to the control group, it is difficult to infer that RJ might be able to normalize these values to the physiological range, which is still not defined due to differences in the techniques used in laboratories. Second, like every meta-analysis, it is possible that the current study is affected by publication bias. Third, considerable heterogeneity was observed across studies, particularly in TAC and hs-CRP analyses. 

The findings of this meta-analysis suggest that RJ supplementation may contribute to enhanced antioxidant capacity and reduced oxidative stress, particularly in non-diabetic individuals and those with normal BMI. These results offer valuable insights into potential health benefits associated with RJ intake. However, more high-quality studies with larger sample sizes and standardized methodologies are necessary to confirm and elucidate the observed effects, taking into account varying dosages and participant characteristics.

The implications of RJ on oxidative stress parameters may pave the way for novel interventions or adjunctive therapies in managing oxidative stress-related conditions. Future research should focus on elucidating the mechanisms underlying these observed effects and exploring the diverse impacts in different population subsets.
